# Cyclopia with proboscis: A rare congenital anomaly

**DOI:** 10.1002/ccr3.4466

**Published:** 2021-07-16

**Authors:** Asma Kunwar, Bibek Man Shrestha, Suraj Shrestha, Pooja Paudyal, Suniti Rawal

**Affiliations:** ^1^ Department of Obstetrics and Gynecology Tribhuvan University Teaching Hospital Kathmandu Nepal; ^2^ Maharajgunj Medical Campus Institute of Medicine Kathmandu Nepal

**Keywords:** congenital defects, cyclopia, holoprosencephaly, proboscis

## Abstract

Cyclopia with a proboscis, a rare congenital anomaly, and a severe form of holoprosencephaly occur as a result of incomplete separation of prosencephalon into two halves of hemispheres during organogenesis. A prenatal anomaly scan can help in the early detection of the condition and timely termination of the pregnancy.

## Graphical abstract

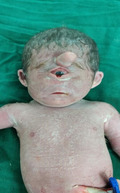

## 1 INTRODUCTION

Cyclopia or alobar holoprosencephaly is a rare and lethal congenital anomaly and a rare form of holoprosencephaly (HPE) which occurs as a result of incomplete separation of prosencephalon into two halves of hemispheres during organogenesis leading to failure of cleavage of orbit into two cavities of eyes.^1^ This entity represents a developmental seize of the anterior terminal of the neural plate. Typically, the nose is either missing or replaced with a nonfunctioning nose in the form of a proboscis.^1^ Such a proboscis that generally appears above the central eye is a characteristic of a form of cyclopia called rhinencephaly or rhinocephaly. While holoprosencephaly affects one in 16,000 live newborns, cyclopia is seen as rarely as one in 100,000 newborns, including stillbirths. Extracranial malformations described in stillbirths with cyclopia include polydactyly, renal dysplasia, and an omphalocele.^2^, ^3^

## 2 CASE REPORT

A 40‐year‐old apparently healthy G_6_P_5+1_L_4_ Tamang, an alcoholic woman with no previous congenital anomalous birth, a homemaker, presented at 31^+^ ^2^ ‐week period of gestation (POG) in the labor room with complaints of lower abdominal pain and decreased fetal movements for 3 days. She never had an antenatal checkup (ANC) in any of her previous pregnancies and neither had any ultra‐sonogram done. She had a spontaneous abortion 4 years back at 12 weeks POG. There was no history of any other drugs or alternative medicine intake, radiation exposure, or history of fever during pregnancy.

**FIGURE 1:**
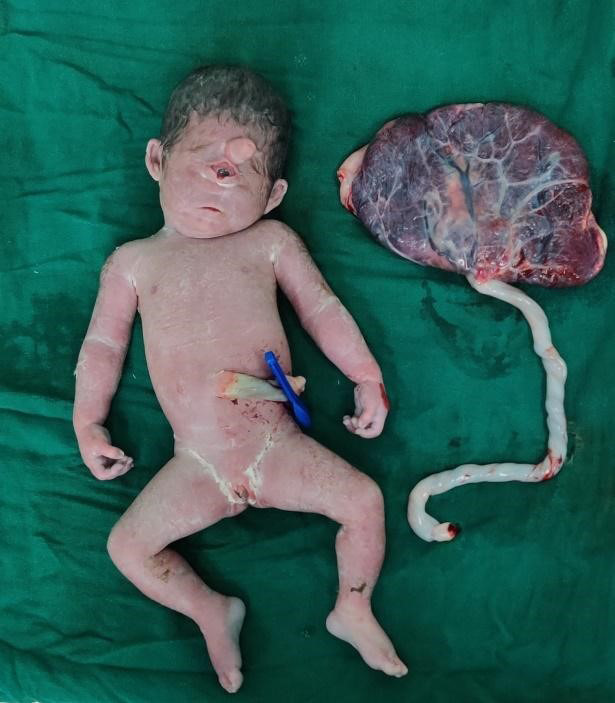
Anomalous baby with normal placenta

**FIGURE 2:**
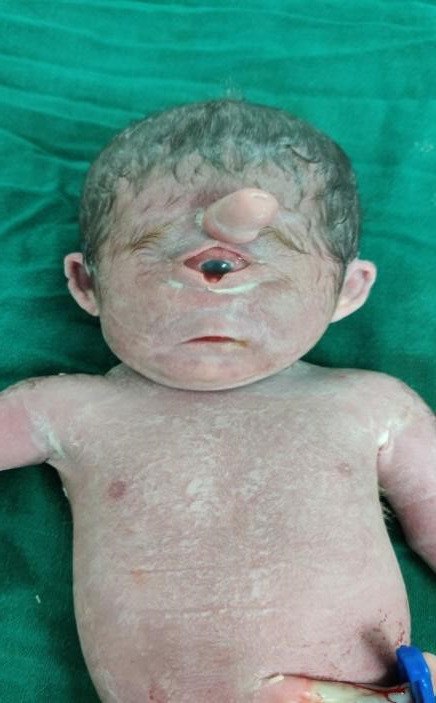
Single eye with proboscis above the eye

On examination, she was conscious with a blood pressure of 120/70 mmHg, respiratory rate of 16/minute and tachycardiac (HR‐120 bpm), and other vitals stable. On abdominal examination, the uterus was of 32‐week size, relaxed; fetal parts were palpable but no fetal heart rate was detected. On vaginal examination, cervical os was 1 cm dilated, soft posterior, 10% effaced, and the amniotic membrane was intact. All her laboratory investigations were within normal limits. Ultrasonography showed a singleton pregnancy of approximately 29^th^‐30^th^ weeks of POG with fundal placenta, amniotic fluid index of 57 cm, cephalic without fetal movement, and cardiac activity; all features were suggestive of intrauterine death with polyhydramnios. There was a spontaneous expulsion of a single dead female fetus of 1.25 kg with a placenta weighing 150 grams with cyclopia with a proboscis. Also, the baby had a dysmorphic face with a single eye and the nose was absent in normal position; however, a nonfunctioning nose was present above the single eye (Figures 1 and 2). There were no other gross congenital anomalies, and the placenta was normal. Histological and biochemical evaluations were not performed.

## 3 DISCUSSION

A cyclopia with proboscis is a rare form of HPE with few more cases reported worldwide.^4^, ^5^, ^6^, ^7^ To our knowledge, this is the first case reported from our institution. Moreover, no case of cyclopia with proboscis has been documented from Nepal to date.

Alobar holoprosencephaly, as reported in this case, represents the most severe form and constitutes undifferentiated cerebral hemispheres with monoventricles, a fusion of thalamus, and other cranial midline abnormalities.^8^, ^9^, ^10^ Most of the cases are sporadic and incompatible with life, and the etiology of this condition remains largely unknown.^11^ However, various heterogeneous risk factors have been implicated. The possible culprits include defective inheritance and environmental factors.^4^ Genes affecting chromosomes 3 and 10, maternal exposure to teratogenic drugs (such as salicylates, amidopyrine, corticosteroids, aspirin, lithium, anticonvulsants, retinoic acid, anticancer agents), alcohol, toxoplasmosis, rubella, cytomegalovirus, and herpes simplex (TORCH) infections, ionic radiation, and maternal diabetes are the notable risk factors in previously reported cases.^4^, ^12^, ^13^, ^14^ As already mentioned, finding an etiologic relation is very difficult in such conditions, mainly due to the limited literature knowledge and our inability to reach the patient's previous laboratory and medical reports, and lack of other aforementioned risk factors, and the regular consumption of alcohol might the associated factor for the fetal anomaly in our case.

A high prevalence of chromosomal abnormalities (mainly trisomy 13) and female predominance in HPE is found. The baby with this abnormality generally gets naturally aborted or is stillborn on delivery.^15^ Our fetus was not genetically tested after the spontaneous expulsion.

Sonography is the most helpful in the prenatal diagnosis of cyclopia, and in most of the reported cases, the anomaly has been recognized early in the anomaly scan. This early diagnosis by fetal ultrasonography allows for timely termination of pregnancy and avoids maternal psychological trauma of giving birth to a deformed fetus.^16^, ^17^ Additionally, fetal MRI can help confirm the sonographic findings and detect any other additional anomaly.^18^ However, in our case, the woman never had any antenatal checkups and the anomaly was diagnosed after the expulsion of the fetus.

Holoprosencephaly is managed supportively along with other associated malformations treatment. Prognosis depends on the degree of fusion and malformation of the brain and other associated complications. Alobar and semilobar HPE have the worst prognosis and are often incompatible with life. However, with lobar HPE, a child can survive for some years with neurological and mental challenges.^19^

This report emphasizes the need for awareness and education about the importance of antenatal checkups and anomalous effects of alcohol to all women of reproductive age groups, particularly in developing countries like Nepal. Our patient was thoroughly counseled about the disease condition and encouraged to take necessary steps in cessation of the alcohol intake and avoid various risk factors.

## 4 CONCLUSION

Cyclopia is a rare congenital anomaly. The importance of an anomaly scan in the timely detection of anomalous babies and preventing mothers from the psychological trauma of carrying such fetus is to be stressed upon to decrease the suffering of parents and family members.

## CONFLICTS OF INTEREST

None to declare.

## AUTHORS CONTRIBUTION

AK, PP, BMS, and SS drafted the manuscript. AK, PP, and SR were involved in editing and revising the manuscript. All the authors read and approved the final version of the manuscript.

## CONSENT FOR PUBLICATION

Written informed consent was obtained from the patient and her husband for publication of this case report and images of the baby. A copy of the written consent is available for review by the Editor‐in‐Chief of this journal.

## Data Availability

All the necessary data and information are within the article.
